# In *vitro* antitumor activity of patulin on cervical and colorectal cancer cell lines

**DOI:** 10.29252/cmm.3.1.25

**Published:** 2017-03

**Authors:** M Abastabar, A Akbari, J Akhtari, MT Hedayati, T Shokohi, H Mehrad-Majd, H Ghalehnoei, S Ghasemi

**Affiliations:** 1 Department of Medical Mycology and Parasitology, Invasive Fungi Research Center (IFRC), Mazandaran University of Medical Sciences, Sari, Iran; 2 Colorectal Research Center, Iran University of Medical Sciences, Tehran, Iran; 3 Immunogenetics Research Center, Department of Medical Nanotechnology, School of Advanced Technologies in Medicine, Mazandaran University of Medical Sciences, Sari, Iran; 4 Clinical Research Unit, Mashhad University of Medical Sciences, Mashhad, Iran; 5 Immunogenetics Research Center, Mazandaran University of Medical Sciences, Sari, Iran; 6 Students Research Committee, Department of Parasitology and Mycology, School of Medicine, Mazandaran University of Medical Sciences, Sari, Iran

**Keywords:** Cancer, HeLa cells, Mycotoxin, Patulin

## Abstract

**Background and Purpose:**

Patulin is a mycotoxin produced by some molds, especially *Aspergillus* and *Penicilium*, and is responsible for mycotoxicosis in animals and humans.

There is still not very detailed data about the anti-cancer potency of patulin, but some reports demonstrated that it induces cellular apoptosis and toxicity.

**Materials and Methods:**

To determine the efficacy of patulin as a therapeutic strategy for cervical and colorectal cancers, we investigated its effects on HeLa,SW-48, and MRC-5 cell lines. Cell lines were exposed to various concentrations of patulin (i.e., 0.5, 1, 2, and 4 µM), then using methyl thiazolyl tetrazolium (MTT) and bromo-2′-deoxyuridine (BrdU) assays, the rates of apoptosis and cell viability were determined.

**Results:**

The obtained results showed a significant reduction in cell viability and apoptosis induction in a dose-dependent manner. Among all the cell lines, the highest growth inhibition rate was obtained at the 4 μM concentration of patulin.

**Conclusion:**

Our results suggested that patulin could significantly decrease tumor growth in human cervical and colorectal cancer models.

## Introduction

Patulin is a toxic secondary metabolite produced by molds, especially *Penicillium* and *Aspergillus*, and is usually found in apples and apple-derived products including juice and compote. Several reports have demonstrated that exposure to different levels of this polyketide metabolite is associated with immunotoxicity, genotoxicity, embryotoxicity, and teratogenicity in animals and some microorganisms [1-6]. 

Regarding many disadvantages of the routine anticancer drugs, researchers are seeking new compounds and formulations with better activities and lower side effects [7, 8]. In this regard, the use of toxins and secreted compounds of fungi and bacteria can be beneficial.

Patulin induces toxicity through two mechanisms, first by creating oxidative stress response in cells and additionally binding to reactive sulfhydryl groups in cellular proteins through covalent bonds [8, 9]. Patulin is known to affect the testis tissue of rats and cause a significant increase in serum testosterone level, but there are no reports on the toxic or apoptotic effects of this toxin on the female reproductive system [10].

Colorectal and cervical cancers are respectively the second and third most frequently diagnosed cancers in women worldwide [11, 12]. In spite of comprehensive understanding regarding the management of various stages of these cancers, definitive treatment remains clinically challenging; thus, finding novel inhibitors and compounds seems to be essential. 

Patulin has been reported to activate apoptosis in several mammalian cell lines, such as human leukemia cells (HL-60), through the generation of reactive oxygen species (ROS) and human embryonic kidney cells 293 (HEK293) by phosphorylation of two major mitogen-activated protein kinases (MAPKs), p38 kinase and c-Jun N-terminal kinase (JNK) [9]. The 3-(4,5-dimethylthiazol-2-yl)-2,5-diphenyltetrazoliumbromide (MTT) assay, a reliable and common colorimetric test for *in vitro* evaluation of cell viability, has been utilized to analyze cell cultures subjected to different doses of patulin [13].

In addition, bromodeoxyuridine (5-bromo-2'-deoxyuridine, BrdU), the analog of the DNA base thymidine, is usually used for the detection of cell apoptosis and proliferation [14]. Therefore, in the present study, HeLa, the human cervical cancer cell line, was treated with different levels of patulin to examine its effect on cell proliferation and toxicity. In addition, we assessed the effect of patulin on two other cell lines, including Medical Research Council cell strain 5 (MRC-5; normal human lung cells) and SW-48 (colonic adenocarcinoma cells), using MTT and BrdU assays.

## Materials and Methods

***Chemicals and cell culture***

The reference powder of patulin, (4-hydroxy-4H-furo[3, 2-c]pyran-2 (6H)-one), was obtained from Sigma Chemical Company (St. Louis, Missouri, USA) and dissolved in 15% ethanol at a concentration of 10 mM and stored at −20°C until use. All other solvents and reagents were used as chemical grade.

Three cell lines, SW-48, HeLa, and MRC-5, were purchased from the National Cell Bank of Iran deposited in Pasteur Institute (Tehran, Iran). All the cell lines were cultured in RPMI 1640 medium (Gibco, Germany) supplemented with 10% fetal bovine serum, 0.1 mg/ml streptomycin, and 100 U/ml penicillin, and then incubated at 37°C in a 5% CO_2_ incubator. 

***Cell viability assay***

The MTT colorimetric assay was used to determine the *in vitro* growth inhibitory effect of patulin on cell lines. The cell lines at a concentration of 5×10^3^ cells/well were seeded in 96-well flat-bottom plates (SPL Life Sciences, South Korea) containing 100 µl of RPMI medium. After 24 h, the cell lines were treated with a serial doubling dilution of patulin. Afterwards, patulin was discarded and the cells received 10 µl of MTT (5 mg/ml stock solution; Roche Applied Science, Germany). All the plates were incubated at 37°C for 4 h, and formazan blue was dissolved in 200 µl of dimethyl sulfoxide (DMSO). Finally, absorbance was measured at a wavelength of 570 nm using a spectrophotometric microplate reader (BioTek Elx 808). All the data are represented as two independent experiments.

***Cell apoptosis assay***

The proliferation of the three cell lines, SW-48, HeLa, and MRC-5, was determined using a colorimetric test based on the evaluation of BrdU (Roche Applied Science, Germany) incorporation during cell division. Briefly, the cells were seeded in plates at a concentration of 5000 cells per insert well, cultured for 24 h to allow the growth of a confluent monolayer, treated with a serial dilution of patulin, and finally, incubated with 10 μM of BrdU for 5 h at 37°C under 5% CO_2_ before analysis. Absorbance values were measured using a spectrophotometric microplate reader (BioTek Elx 808) at 490 nm. All the tests were performed in duplicate.

Growth inhibition rate and cell proliferation were calculated by using the following formula:

Growth inhibition rate % = 1− (OD _drug exposure _/ OD _control_ ) _˟_ 100

Cell proliferation % = 1− (OD _drug exposure _/ OD _control _) _˟_ 100(7)

***Statistical analysis***

IC50 of formulations were calculated using CalcuSyn version 2 software (BIOSOFT, UK). Differences in inhibition and proliferation were analyzed using Student’s t-test. *P-values* less than 0.05 were considered statistically significant. 

## Results

To determine the growth inhibitory effect of patulin, all the cell lines, SW-48, HeLa, and MRC-5, were treated with various concentrations of patulin (i.e., 1 μM, 2 μM, 4 μM, and 8 μM) for 24 h and cell viability and proliferation were determined by MTT and BrdU assays and finally presented as IC50 and proliferation rate. 

**Figure 1:**
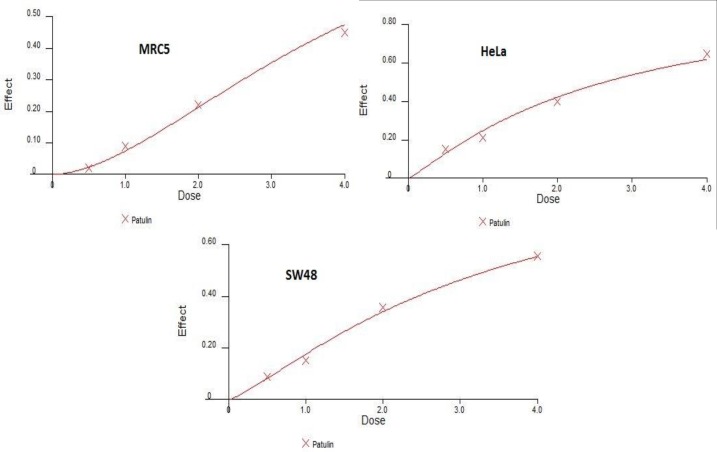
Assessment of *in vitro* growth inhibition by MTT test. Results revealed significant changes in the treated SW-48, HeLa, and MRC-5 cell lines versus the untreated ones (*P<0.05*).

The results of MTT and BrdU assays indicated that patulin had cytotoxic effects on all the cell lines in a dose-dependent manner. As illustrated in Figure 1, assessment of *in vitro* growth inhibition by MTT assay revealed significant changes in the treated SW-48, HeLa, and MRC-5 cell lines versusthe untreated cells (*P<0.05*). Patulin caused dose-dependent induction of cytotoxicity at the concentrations of 0.5, 1, 2, and 4 μM after 24 h of treatment.

**Figure 2:**
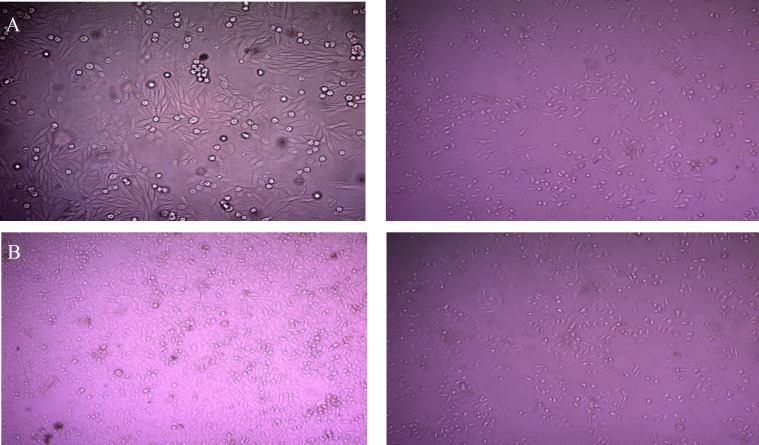
Effects of patulin on morphology of SW48 (A) and HeLa (B) cells by BrdU assay; right: untreated cell, left: cells after treatment by patulin

Figure 2 demonstrated that the treatment of HeLa and SW-48 cell lines with patulin resulted in reduction in cell numbers. 

The highest IC50 values for the SW-48, HeLa, and MRC-5 cell lines, as indicated by the MTT assay, were 55%, 65%, and 45%, respectively, at a concentration of 4 μM (Table 1).

As presented in Figure 1, the cell proliferation rates of SW-48 and HeLa cells decreased in a dose-dependent manner. The lowest cell proliferation rates of SW-48, HeLa, and MRC-5, as revealed by the 

**Table 1:** Effects of patulin on growth of SW-48, HeLa, MRC5 cell lines

**MTT assay**			
** Inhibition rate (IR)%**			
**SW-48**	**HeLa**	**MRC5**	**Concentration of patulin (μM)**
8	15	2	0.5
15	21	9	1
30	40	22	2
55	65	45	4

BrdU assay, were 29%, 17%, and 30%, respectively, at a concentration of 4 μM (Table 2).

**Table 2:** Effects of patulin on growth of SW-48, HeLa, MRC5 cell lines

**BrdU assay**			
** Cell proliferation%**			
**SW-48**	**HeLa**	**MRC5**	**Concentration of patulin (μM)**
70	97	87	0.5
49	52	45	1
41	32	37	2
29	17	30	4

## Discussion

Currently, the incidence of colon and cervical cancers is increasing worldwide and are the third and fourth leading causes of female mortality, respectively [15, 16]. In spite of advances in the treatment of these cancers in recent years, the therapeutic strategies pose various challenges to specialists, such as unwanted side effects, incompliancy of patients, as well as development of metastasis and resistance to different drugs [17-19]. Therefore, it is essential to look for potential efficient compounds to design novel drugs targeting colon and cervical cancers.

Patulin is a toxic substance produced by some fungi that has potent anticancer activity; however, its exact biological target remains inconspicuous [1, 6]. Rare reports have been published on the effects of patulin on human cell lines, particularly tumor cells.

In this study, the anti-tumor effects of patulin on a colonic adenocarcinoma cell line (SW-48) and a cervical cancer cell line (HeLa) in an *in vitro* model (MTT and BrdU assay) were evaluated. Our results demonstrated that treatment with patulin significantly prevented the growth of tumors, as revealed in figures 1 and 2, as well as tables 1 and 2.

Previous studies revealed that patulin induced toxicity in human cells including human embryonic kidney 293 (HEK293) [9] and human immortalized keratinocyte (HaCaT) [20], as well as animal cells such as Chinese hamster ovary (CHO-K1) [13], V79 Chinese hamster cells [21], and spontaneously immortalized rat granulosa cells (SIGC) [22]. In a study by Wu et al., the lowest cell viability and greatest morphological changes of HL-60 cells were observed at the concentration of 2.5 μM [8]. 

In the current study, MTT assay demonstrated that the inhibition rates of HeLa and SW-48 cell lines, which were incubated with 2 μM of patulin, were 40% and 30%, respectively (Table 1). Patulin at a concentration of 4 μM also reduced the viability of tumor cells to 55% for SW-48 cell lines and 65% for HeLa cell lines, respectively.

However, a study by Luft et al. (23), it was reported that the treatment of peripheral blood mononuclear cells (PBMCs) with a lower concentration of patulin (0.6 μM) resulted in the reduction of cell viability. The results of this study (Table1) indicated that cell viability reduced in MRC-5, normal cell lines of the lungs, which is in agreement with the findings of Liu et al. [9] who found that patulin reduced cell viability in HEK cells. 

It is worth mentioning that MTT assay indirectly quantifies cell number and cannot obviously confirm the induction of apoptosis and reduced cell number; thus, the apoptotic effect of patulin on tumor cell growth was evaluated using an ELISA-based BrdU incorporation assay.

Several studies have confirmed that patulin can induce apoptosis in various cell lines [9]. Wu et al. reported that apoptosis was initiated by patulin in intrinsic pathway by the Bcl-2 family, in which cytochrome c is released into cytosol and the level of cleaved caspase-9 is elevated [24]. Additionally, the activation of p38 kinase and c-Jun N-terminal kinase signaling was introduced as apoptotic pathways in HEK cells induced by patulin [9]. Exposure of HeLa and SW-48 cell lines to patulin led to the induction of apoptosis as presented in BrdU assay (Table 2 and Figure 2). As presented by BrdU assay, the best apoptotic effect on all the cell lines was obtained at the concentration of 4 μM of patulin, and among all the cells lines, the rate of apoptosis was the highest in HeLa.

Previously, BrdU has been used as a rapid test to determine DNA synthesis in proliferating cells exposed to different mycotoxins. In the study of Nagashima et al. using this assay, the IC50 value of nivalenol against the promyelocyte-derived cell line, HL60, was 0.51 µM [25]. Also, fusarochromanone, a mycotoxin produced by *Fusarium equiseti*, exhibited IC50 values ranging from 10 nM to 2.5 μM against HaCat, P9-WT, MCF-7, MDA-231, SV-HUC, UM-UC14, and PC3 cell lines [26]. 

The current results regarding patulin are in line with those obtained by Wu and Liu, who concluded that patulin induced apoptosis in HL-60 and HEK293 cells as well as similar to other mycotoxins [25, 26].

## Conclusion

We believe that because of verified induction of toxicity and apoptosis in our report and other studies, patulin can be considered an extreme candidate for anti-cancer agents. Fungus usually produces many important metabolites that may have anti-cancer benefits. For the first time we have demonstrated that patulin was able to decrease tumor growth in human colorectal and cervical cancer models. 
